# Surufatinib plus toripalimab combined with etoposide and cisplatin as first-line treatment in advanced small-cell lung cancer patients: a phase Ib/II trial

**DOI:** 10.1038/s41392-024-01974-2

**Published:** 2024-09-27

**Authors:** Yaxiong Zhang, Yan Huang, Yunpeng Yang, Yuanyuan Zhao, Ting Zhou, Gang Chen, Shen Zhao, Huaqiang Zhou, Yuxiang Ma, Shaodong Hong, Hongyun Zhao, Li Zhang, Wenfeng Fang

**Affiliations:** 1 Department of Medical Oncology, State Key Laboratory of Oncology in South China, Guangdong Provincial Clinical Research Center for Cancer, Collaborative Innovation Center for Cancer Medicine, Sun Yat-sen University Cancer Center, Guangzhou, China; 2 Department of Clinical Research, State Key Laboratory of Oncology in South China, Guangdong Provincial Clinical Research Center for Cancer, Collaborative Innovation Center for Cancer Medicine, Sun Yat-sen University Cancer Center, Guangzhou, China

**Keywords:** Lung cancer, Drug development

## Abstract

There is still room for improvement in first-line treatment of advanced small cell lung cancer (SCLC). This trial firstly investigated efficacy and safety of antiangiogenic therapy (surufatinib) (200 mg, qd, po) plus anti-PD-1 treatment (toripalimab) (240 mg, d1, ivdrip) combined with etoposide (100 mg/m², d1-d3, iv, drip) and cisplatin (25 mg/m², d1-d3, ivdrip) for advanced SCLC as first-line treatment, which has been registered on ClinicalTrials.gov under the identifier NCT04996771. The four-drug regimen was conducted q3w for 4 cycles with maintenance therapy of surufatinib and toripalimab. The primary endpoint was progression-free survival (PFS). The secondary end points included objective response rate (ORR), disease control rate (DCR), overall survival (OS) and safety. All of the 38 patients were enrolled for safety analysis, while only 35 patients were enrolled for efficacy analysis since loss of efficacy evaluation in 3 cases after treatment. After a median follow-up of 21.3 months, the ORR was 97.1% (34/35), and the DCR and the tumor shrinkage rate were both 100% (35/35). The median PFS was 6.9 months (95% CI: 4.6 m–9.2 m) and the median OS was 21.1 months (95% CI: 12.1 m–30.1 m). The 12-month, 18-month, and 24-month OS rates were 66.94%, 51.39% and 38.54%. The occurrence rate of grade ≥3 treatment-emergent adverse events (TEAEs) was 63.2% (24/38), including neutrophil count decreased (31.6%, 12/38), white blood cell count decreased (23.7%, 9/38) and platelet count decreased (10.5%, 4/38). No unexpected adverse events occurred. This novel four-drug regimen (surufatinib, toripalimab, etoposide plus cisplatin) revealed impressive therapeutic efficacy and tolerable toxicities.

## Introduction

The main cause of morbidity and mortality in malignant tumor is lung cancer.^1^ There are approximately 2.2 million incidental lung cancer cases globally in 2020, with about 13–15% small cell lung cancer (SCLC) accounts for the contribution.^2^

Tobacco smoking is the leading risk factor for SCLC which accounts for ≥ 95% of the whole population in SCLC patients.^3^ SCLC is a kind of neuroendocrine tumor (NET) clinically characterized by a rapid growth and early distant metastasis.^3^ It is usually diagnosed at the extensive stage (ES) with prognosis poor.^3^ As a highly aggressive subtype of lung NET, SCLC is characterized by deletion of multiple tumor suppressor genes, such as mutations in TP53 and RB1.^4^ This characteristic also determines that SCLC is difficult to be treated with target therapy precisely. Traditional chemotherapy has always been unable to bring significant survival benefits to ES-SCLC patients.^5^ In the past 30 years, etoposide combined with cisplatin/carboplatin has been the most commonly used therapeutic regimen for treatment-naïve ES-SCLC. The objective response rate (ORR) of this platinum–etoposide doublet regimen ranges from 44% to 78%, with about 4–5 months as the median progression-free survival (mPFS) and 10 months as the median overall survival (mOS).^6^

With the development of immunotherapy, ES-SCLC has ushered in a breakthrough in first-line treatment.^7^ Multiple phase III randomized controlled clinical studies have proved that the efficacy of anti-programmed death-1 (PD-1)/programmed death ligand-1 (PD-L1) inhibitors, such as atezolizumab, durvalumab, serplulimab, adebrelimab, tislelizumab or toripalimab, combined with etoposide plus cisplatin/carboplatin is significantly better than platinum–etoposide doublet regimen. The mPFS is still about 4–5 months, while the mOS has increased from 10 months to about 12–15 months.^8–13^ Although the results of these trials are encouraging, the curative effect of the combination regimen of immunotherapy and chemotherapy in SCLC seems to be far inferior to that in NSCLC,^14,15^ possibly due to the low-level expression of PD-L1 and immunosuppression in SCLC.^16,17^ Recent studies have revealed that although SCLC has a high-level tumor mutational burden (TMB), it still belongs to the immunosuppressive phenotype due to a low level of tumor-infiltrating lymphocytes.^18^ Despite the positive results of chemotherapy plus immunotherapy in multiple clinical trials have indeed improved the current treatment status for advanced SCLC,^8–13^ there is still room for improvement in first-line treatment.

Surufatinib is a novel oral angio-immuno tyrosine kinase inhibitor (TKI) with multiple targets including vascular endothelial growth factor receptors (VEGFR) 1, 2, and 3, fibroblast growth factor receptor type 1 (FGFR1) and colony-stimulating factor-1 receptor (CSF-1R).^19^ It is the first targetable therapeutic drug approved globally to treat NET from all sources of organs, including lung.^19^ There is evidence that VEGFR, FGFR, and CSF-1R play important roles in the regulations of tumor immunity. Tumor cells activate the VEGFR signaling pathway on T cells by secreting VEGF, thereby reducing the anti-tumor activity of T cells.^20^ It has also been confirmed that inhibition of FGFR and CSF-1R can induce the proliferation and differentiation of tumor-associated macrophages (TAM), thereby mediating the regulation of tumor immunity.^21^ Preclinical study revealed that surufatinib could significantly decrease the infiltration of CSF-1R positive M2 TAMs into tumor tissues and increase the infiltration of M1-TAMs and CD8 positive T cells.^22^ In vivo study also showed that combination of surufatinib with anti-PD-L1 could improve the inhibition of tumor growth and prolong the survival.^22^ Recent trial has explored the efficacy of surufatinib plus toripalimab, an anti-PD-1 inhibitor, as the second-line treatment of ES-SCLC, which exhibited the synergistic effect of surufatinib in combination with immune checkpoint inhibitors. It showed that surufatinib plus toripalimab could achieve about 3 months of the mPFS and 10.94 months of the mOS, with 95% of disease control rate (DCR) in patients with advanced SCLC after failure of front-line systemic chemotherapy.^23^ The combination of surufatinib and toripalimab has shown potentially efficacy as subsequent treatment of SCLC. Besides, cisplatin could decrease the expression of glutathione peroxidase 4 (GPX4), which was crucial for antioxidant defense in cells, while surufatinib could increase the expression of acyl-CoA synthetase long-chain family member 4 (ACSL4), which is critical for lipid peroxides accumulation. In vitro assays revealed that surufatinib could exert a pronounced synergistic antitumor effect on SCLC cell lines by inducing ferroptosis when combined with cisplatin.^24^ It is justified for incorporating surufatinib into immunotherapy and chemotherapy to enhance anti-tumor effect.

Based on the basic researches and clinical data above, we proposed the research hypothesis that antiangiogenic therapy (surufatinib) plus anti-PD-1 treatment (toripalimab) combined with platinum–etoposide doublet regimen could further improve the response rate and survival time of ES-SCLC patients. Therefore, we conducted this prospective, single-arm, trial to investigate the efficacy of surufatinib plus toripalimab combined with etoposide and cisplatin for advanced SCLC as first-line treatment, as well as exploring the safety and tolerability of this four-drug regimen.

## Results

### Patient enrollment and baseline characteristics

**Fig. 1:**
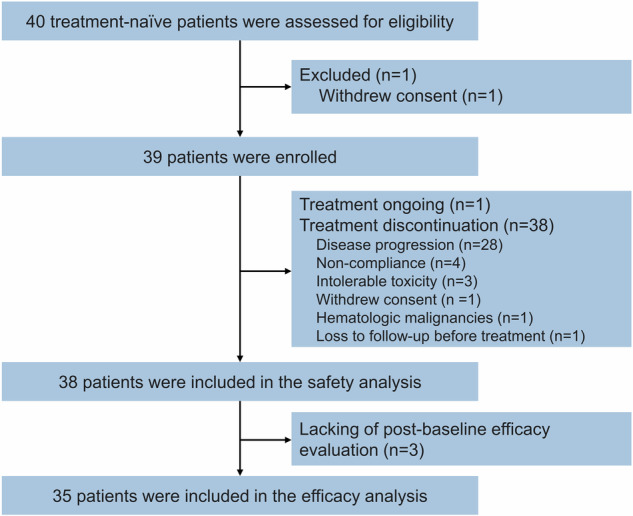
Patient flowchart

This study recruited 39 patients (phase Ib, *n* = 6; phase II, *n* = 33) between December 2021 and August 2023. Two suspected dose-limiting toxicities (DLTs) were observed in the 6 patients (one of the first 3 patients, and one of the subsequent 3 patients) in the dose-escalation phase for the 1st cycle. The first DLT was myelosuppressive toxicity (white blood cell count decreased, neutrophil count decreased, both were grade 4), and the other was treatment unrelated to incomplete intestinal obstruction in grade 3. Considering that the myelosuppressive DLT was mainly associated with chemotherapy and both of the above DLTs recovered soon after symptomatic treatment, as well as tolerable toxicities of 200/250 mg, qd, po. of surufatinib in relevant research focused on four-drug regimens of surufatinib combined immunochemotherapy,^25,26^ the investigators finally decided not to reduce or increase the initial dose and regarded the recommended phase 2 dose (RP2D) of surufatinib as 200 mg, qd, po. 38 patients received at least one cycle therapy were enrolled for safety analysis, while only 35 patients were enrolled for efficacy analysis since 3 cases without efficacy evaluation in after treatment (Fig. 1). Patients with a median age of 64 (range: 21–74) years were predominantly men (82.1%, 32/39), with smoking history (64.1%, 25/39), with an Eastern Cooperative Oncology Group (ECOG) performance status (PS) score of 1 (59.0%, 23/39), had stage IV disease (89.7%, 35/39), initially diagnosed as ES-SCLC (87.2%, 34/39). The most common distant metastatic organs of these enrolled patients were liver (33.3%, 13/39) and bone (30.8%, 12/39). Table 1 summarized the baseline characteristics of the enrolled participants. The median time of follow-up was 21.3 months, ranged from 4.6 months to 28.9 months, at the last time of data collection (Aug. 9, 2024).

**Table 1:** Baseline characteristics of patients

Characteristics	*n* = 39
**Age, years, median (range)**	64 (21–74)
≥65	18 (46.2)
<65	21 (53.8)
**Gender, n (%)**	
Female	7 (17.9)
Male	32 (82.1)
**ECOG performance status, n (%)**	
0	16 (41.0)
1	23 (59.0)
**TNM staging at screening, n(%)**	
IIIB	4 (10.3)
IV	35 (89.7)
**Smoking history, n(%)**	
Never	14 (35.9)
Current/Former	25 (64.1)
**Distant metastases, n(%)**	
Liver	13 (33.3)
Bone	12 (30.8)
Kidney	8 (20.5)
Brain	5 (12.8)
Other^a^	4 (10.3)
**Disease stage at diagnosis, n (%)**	
Extended	34 (87.2)
Limited	5 (12.8)

^a^3 patients with pancreas metastases; 1 patient with neck metastases. ECOG: Eastern Cooperative Oncology Group

### Efficacy

**Fig. 2:**
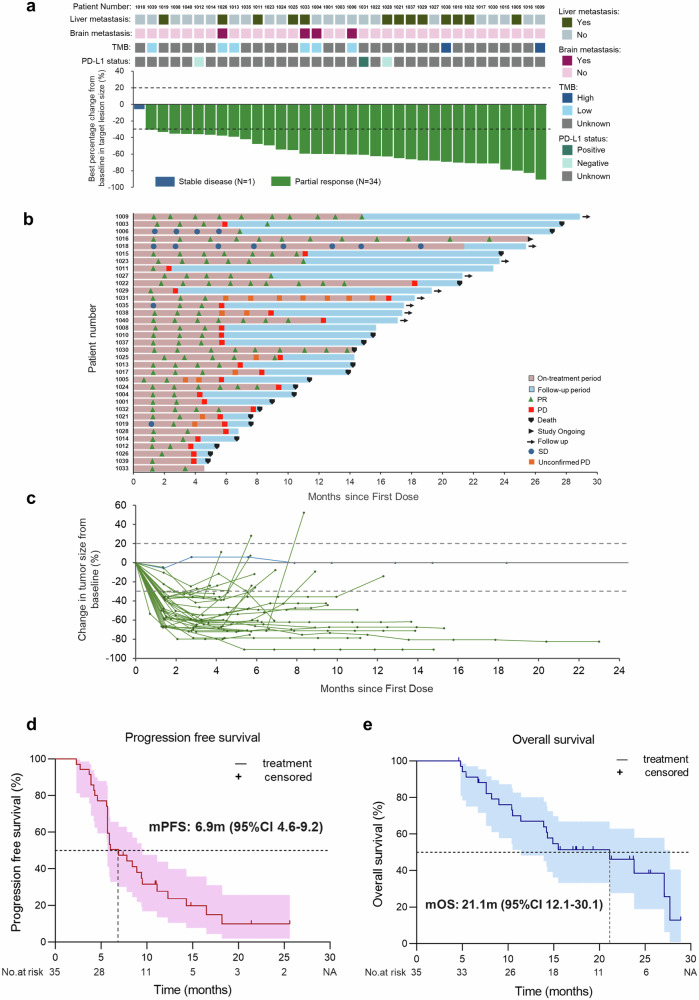
Treatment response and survival analysis. **a** Best percentage changes from baseline in target lesions; **b** Treatment exposure and response duration; **c** Change of tumor size overall time. **d** Kaplan–Meier curves of progression-free survival; **e** Kaplan–Meier curves of overall survival

The ORR was 97.1% (34/35) and DCR was 100% (35/35). Tumor shrinkage was achieved in 100% of patients (Table 2 and Fig. 2a–c). The mPFS was 6.9 months (95% CI: 4.6 m–9.2 m) in overall 35 evaluable patients (Fig. 2d), with the 6-month PFS rate of 50.44% and the 12-month PFS rate of 27.69% (Table 2). The mOS was 21.1 months (95% CI: 12.1 m–30.1 m) (Fig. 2e), with the 12-month OS rate of 66.94%, the 18-month OS rate of 51.39% and the 24-month OS rate of 38.54% (Table 2). Duration of response (DOR) events occurred in 23 of 34 patients and the mDOR was 5.0 months (95% CI: 3.6 m–6.4 m) (Supplementary Fig. 1). 32 patients received maintenance therapy with a mPFS of 7.8 months (95% CI 3.8 m–11.8 m), while the mPFS of maintenance therapy was 5.0 months (95% CI 0.6 m–9.4 m) (Supplementary Fig. 1). Subgroup analysis revealed that patients without liver metastases showed significantly longer mPFS (9.4 m vs. 5.7 m, *p* = 0.0053) compared with those with liver metastases (Supplementary Fig. 2). In addition, longer mPFS was also observed in patients without bone metastases (8.4 m vs. 5.8 m, *p* = 0.1927) or without brain metastases (6.9 m vs 5.9 m, *p* = 0.5357) (Supplementary Fig. 2). Supplementary Fig. 3 revealed univariate Cox regression analysis of PFS in detail. More than fifty percent (54.28%, 19/35) of the patients enrolled in the efficacy analysis received the subsequent therapy, including chemotherapy, antibody-drug conjugates (ADCs), VEGFR-TKIs and immune checkpoint inhibitors (ICIs) (Supplementary Fig. 4). Patients received subsequent therapy had significantly longer post progression survival (PPS) (9.2 m vs 1.7 m, *p* = 0.0002) compared with those with no subsequent therapy (Supplementary Fig. 5).

**Table 2:** Tumor response

Variables	*N* = 35
Best objective response, n (%)	
Complete response	0
Partial response	34 (97.1)
Stable disease	1 (2.9)
Progressive disease	0
Objective response rate, n (%)	34 (97.1)
95% CI	(85.5, 99.5)
Disease control rate, n (%)	35 (100)
95% CI	(90.1, 100)
TTR, months, median (95% CI)	1.4 (1.36, 1.44)
DOR, months, median (95% CI)	5.0 (3.6, 6.4)
PFS, months, median (95% CI)	6.9 (4.6–9.2)
6-month PFS rate, %	50.44
12-month PFS rate, %	27.69
OS, months, median (95% CI)	21.1 (12.1–30.1)
12-month OS rate, %	66.94
18-month OS rate, %	51.39
24-month OS rate, %	38.54

*TTR* time to response, *DOR* duration of response, *PFS* progression free survival, *OS* overall survival, *CI* confidence interval

### Biomarker analysis

**Fig. 3:**
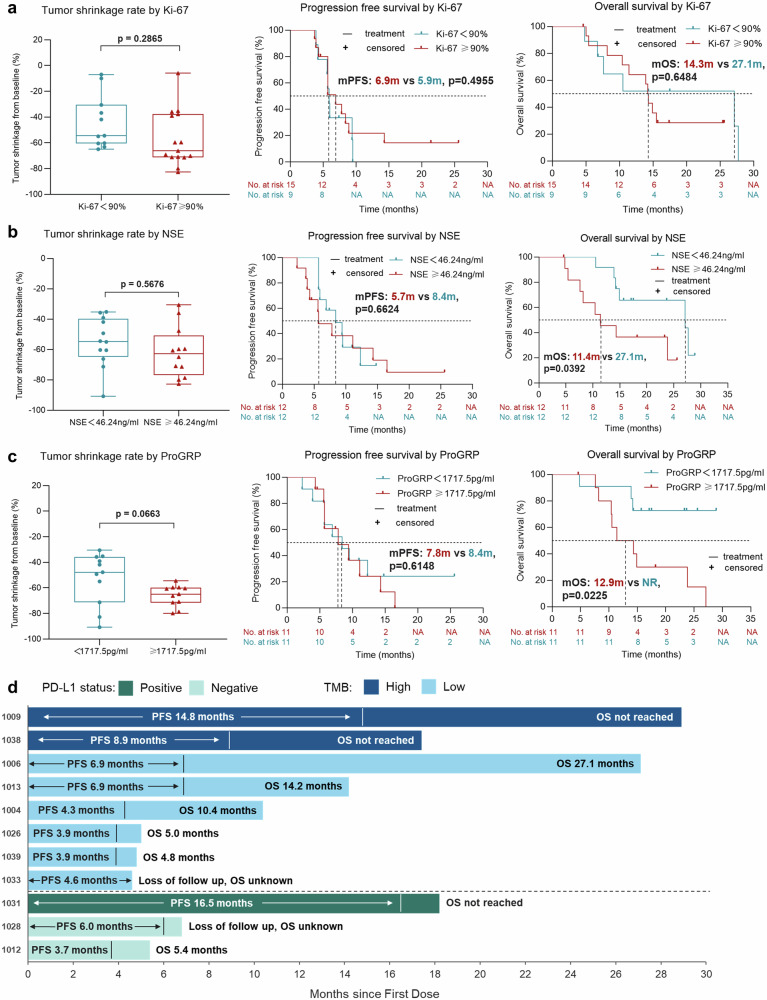
Biomarker analysis stratified by the baseline levels of Ki-67, NSE, ProGRP, and the status of PD-L1 expression & TMB. Tumor shrinkage rate and Kaplan–Meier curves of PFS & OS stratified by the baseline levels of **a** Ki-67, **b** NSE, **c** ProGRP. **d** Swimmer plot stratified by the status of PD-L1 expression and TMB at baseline

The baseline Ki-67 levels of tumor tissue had no significant influence on tumor shrinkage rate, PFS and OS (Fig. 3a). Additionally, the levels of serum neuron specific enolase (NSE) & pro-gastrin-releasing peptide (ProGRP) also had no implication on tumor shrinkage rate and PFS (Fig. 3b, c). However, patients with low-level NSE or ProGRP had significantly longer OS (27.1 m vs 11.4 m, *p* = 0.0392; NR vs 12.9 m, *p* = 0.0225) compared with high-level NSE or ProGRP (Fig. 3b, c). After carefully recollecting the data of common biomarker associated with immunotherapy response at baseline, we found that PD-L1 expression was tested in only 3 patients (PD-L1 positive, *n* = 1; PD-L1 negative, *n* = 2), while next-generation sequencing (NGS) was performed in 8 patients (TMB-high, *n* = 2; TMB-low, *n* = 6) without overlap. All of the above 11 patients had partial response with a best tumor shrinkage from −30.6% to −90.8% (Supplementary Table 1). The PD-L1 positive case had a 16.5-month PFS, while the PFS of two PD-L1 negative cases were 3.7 month and 6.0 month respectively. The PFS of two TMB-high patients were 8.9 month and 14.8 month respectively, while the mPFS was 4.5 months in 6 TMB-low patients. It exhibited similar trends that PD-L1 positive or TMB-high patients had numerically longer OS compared to PD-L1 negative or TMB-low cases (Supplementary Table 1 and Fig. 3d).

### Safety

All patients (100%, 38/38) experienced ≥1 treatment emergent adverse events (TEAEs), while 24 patients (63.2%, 24/38) experienced grade ≥3 TEAEs. The top five most common ( ≥ 20%) TEAEs were anemia (68.4%, 26/38), proteinuria (63.2%, 24/38), decreased white blood cell count (52.6%, 20/38), hair loss (52.6%, 20/38) and diarrhea (50.0%, 19/38). Besides, the top five grade ≥3 TEAEs were neutrophil count decreased (31.6%, 12/38), white blood cell count decreased (23.7%, 9/38), platelet count decreased (10.5%, 4/38), anemia (7.9%, 3/38) and diarrhea (5.3%, 2/38). No unexpected adverse events occurred. The detailed TEAEs were illustrated in Table 3. 12 SAEs were observed, and detailed information was shown in Supplementary Table 2.

**Table 3:** Treatment emergent adverse events of all grades occurring in more than 15% of patients

Events, n (%)	All patients (*n* = 38)		
	Any grade	Grade 1–2	Grade 3 or higher
All events	38 (100)	38 (100)	24 (63.2)
Anemia	26 (68.4)	23 (60.5)	3 (7.9)
Proteinuria	24 (63.2)	24 (63.2)	0
White blood cell decreased	20 (52.6)	11 (28.9)	9 (23.7)
Alopecia	20 (52.6)	20 (52.6)	0
Diarrhea	19 (50.0)	17 (44.7)	2 (5.3)
Blood creatinine increased	17 (44.7)	17 (44.7)	0
Neutrophil count decreased	16 (42.1)	4 (10.5)	12 (31.6)
Inappetence	16 (42.1)	16 (42.1)	0
Pain	16 (42.1)	16 (42.1)	0
Platelet count decreased	15 (39.5)	11 (29.0)	4 (10.5)
Fatigue	14 (36.8)	14 (36.8)	0
Dizziness	13 (34.2)	13 (34.2)	0
Vomit	12 (31.6)	12 (31.6)	0
Astriction	12 (31.6)	12 (31.6)	0
Serum uric acid increased	12 (31.6)	12 (31.6)	0
Aspartate aminotransferase increased	11 (28.9)	11 (28.9)	0
Nausea	11 (28.9)	11 (28.9)	0
Cough	11 (28.9)	10 (26.3)	1 (2.6)
Alanine aminotransferase increased	9 (23.7)	9 (23.7)	0
Rash	9 (23.7)	9 (23.7)	0
Aypnia	8 (21.1)	8 (21.1)	0
Hypertension	7 (18.4)	6 (15.8)	1 (2.6)
Fever	7 (18.4)	7 (18.4)	0
Creatinine clearance decreased	7 (18.4)	6 (15.8)	1 (2.6)
Dyspnea	7 (18.4)	7 (18.4)	0
Infection	7 (18.4)	7 (18.4)	0
Free thyroxine decreased	7 (18.4)	7 (18.4)	0
Thyroid stimulating hormone increased	6 (15.8)	6 (15.8)	0
Triglycerides increased	6 (15.8)	6 (15.8)	0

## Discussion

Our study explored the therapeutic effect and safety of antiangiogenic therapy (surufatinib) plus anti-PD-1 treatment (toripalimab) combined with platinum–etoposide doublet regimen as first-line treatment of ES-SCLC for the first time. The order of administration is as follows: toripalimab, etoposide, cisplatin, and surufatinib. The reason why immunotherapy is given prior to chemotherapy might be that immunotherapy given earlier could mobilize immune cells to the fullest extent, to avoid destruction of immune cells caused by chemotherapy indiscriminately. However, there is no consensus regarding the sequence of immunotherapy and chemotherapy. Further basic and clinical research is warranted. The reason why etoposide is administered before cisplatin might be related to the characteristics of small cell lung cancer: high degree of malignancy with rapid tumor growth. Etoposide, a topoisomerase II inhibitor, and a cell cycle-specific drug, is given earlier to kill a large number of tumor cells at the proliferative stage to reduce the tumor burden. Thereafter, cisplatin, a cell cycle non-specific drug, is administered to kill the residual tumor cells at other stages. In this combined regimen of four drugs, the optimal administration sequence of surufatinib (before or after immunochemotherapy) is still unclear, which is worthy of later exploration.

The initial dose of surufatinib combined with toripalimab, etoposide plus cisplatin in 3 + 3 dose escalation phase was set at 200 mg qd, po, in order to maximize the survival benefits for patients in a potential tolerable dose. Two suspected DLTs were observed in the 6 patients in the dose-escalation phase for the 1st cycle. The first DLT was myelosuppressive toxicity, the other was treatment unrelated to incomplete intestinal obstruction in grade 3. Based on the 3 + 3 dose escalation principle, the dose of surufatinib should theoretically be reduced to 150 mg, qd, po. The myelosuppressive DLT was mainly associated with chemotherapy and both of the above DLTs recovered soon after symptomatic treatment. Moreover, several studies focused on four-drug regimens of surufatinib combined immunotherapy and doublet chemotherapy have shown tolerable toxicities of surufatinib in 200 mg qd or 250 mg qd.^25,26^ After taking the above into consideration to maximum the efficacy in a potential tolerable dose, the investigators cautiously decided not to reduce the initial dose of surufatinib and take 200 mg qd as preliminary RP2D tentatively. Then, we enrolled more patients in this dose and monitored safety closely. The final results revealed that 63.2% patients (24/38) experienced grade ≥3 treatment emergent adverse events (TEAEs) which was similar to that in the previous study (IMpower 133).^8^ It suggested that surufatinib (200 mg qd) was tolerable combined with toripalimab, etoposide plus cisplatin and could be regarded as the optimal therapeutic dose.

Meanwhile, the ETER701 study recently presented at the 2023 WCLC meeting compared the efficacy and safety of anlotinib plus benmelstobart (an anti-PD-L1 inhibitor) combined with chemotherapy to placebo plus chemotherapy as the first-line treatment of ES-SCLC.^27^ The results showed that the mOS and mPFS of the above four-drug regimen reached 19.3 months and 6.9 months with 81.3% of ORR, which were significantly better than chemotherapy alone.^27^ Our results showed that surufatinib plus toripalimab combined with etoposide and cisplatin had superior short-term efficacy with 97.1% of ORR and 100% of DCR, indicating that the four-drug regimen could provide rapid and deep tumor shrinkage. With a mPFS of 6.9 months and a mOS of 21.1 months, it exceeded the current efficacy data of the first-line treatment in advanced SCLC in published trials,^8–13,27^ showing certain therapeutic advantages potentially. The most common TEAEs in our trial were anemia (68.4%), proteinuria (63.2%), decreased white blood cell count (52.6%), hair loss (52.6%) and diarrhea (50.0%). Grade 3 or higher TEAEs were also controllable. Moreover, the proportions of severe TRAEs of the four-drug regimen in ETER701^27^ were higher than those in our study. It was proved that our four-drug regimen has a good safety profile and can be tolerated by most patients.

Our study firstly explored the four-drug regimen containing surufatinib for treatment-naive patients with ES-SCLC and it was the first study combined small molecule anti-angiogenic TKI plus anti-PD-1 antibody and platinum–etoposide doublet regimen for ES-SCLC, while the ETER701 study^27^ investigated antiangiogenic therapy plus anti-PD-L1 antibody and doublet chemotherapy for ES-SCLC. Both of our trial and ETER701 study indicated that traditional chemotherapy combined with antiangiogenic therapy plus immunotherapy might become the standard regimen as the first-line treatment for ES-SCLC. At present, there are many kinds of anti-PD1/PD-L1 drugs, as well as sorts of antiangiogenic regimens including macromolecular antibodies and micromolecular TKIs. It is necessary to further optimize the selection in combination treatment of antiangiogenic therapy and immunotherapy in the future. Besides, considering the superior efficacy of this novel four-drug regimen in SCLC, it indicated that anti-tumor angiogenesis combined with immunochemotherapy might be a potential direction for other solid tumors and worthy of further exploration.

This novel trial on anti-tumor angiogenesis combined with immunochemotherapy in advanced SCLC patients also had some limitations. First, the total sample size of this single-arm trial was restricted. It was difficult to make a definite decision due to a lack of control arm. Moreover, the selection of the RP2D was questionable due to the small sample size, which might undercut the statistical validity of the findings. A large randomized controlled study with much more enrolled patients is required to identify the superior role of the novel four-drug regimen in SCLC. In addition, we conducted this study without pre-collection of specimens from enrolled patients so we had to analyze potential biomarkers associated with treatment response through post hoc analysis as far as possible. After a full review of electronic medical records, we only recollected data of PD-L1 expression and TMB from 11 enrolled patients for biomarker analysis, as a result of PD-L1 expression and NGS were not routine for SCLC. It seemed that PD-L1 positive or TMB-high patients might get more benefit from four-drug regimen, but the biomarker analysis for treatment response is still insufficient. Tumor samples should be collected prospectively at baseline for more complete biomarker analysis in subsequent trials to validate the above hint.

In conclusion, our study firstly explored the efficacy and safety of antiangiogenic therapy (surufatinib) plus anti-PD-1 treatment (toripalimab) combined with etoposide and cisplatin for ES-SCLC patients as first-line treatment. This novel four-drug regimen revealed impressive therapeutic efficacy and tolerable toxicities.

## Materials and methods

### Patients

Patients who were treatment-naïve with unresectable stage IIIB-IV ES-SCLC could be enrolled. Additional main inclusion criteria included: age ≥ 18 years, ECOG PS 0–1, normal organ function, and at least one of measurable disease according to the Response Evaluation Criteria in Solid Tumors version 1.1 (RECIST v1.1). Recurrent SCLC patients who received previous radical chemoradiotherapy could be included with a treatment-free interval of at least 6 months from the end of treatment. The critical exclusion criteria include previously received anti-PD-1/PD-L1 or antiangiogenic therapy, the presence of unstable or clinically symptomatic brain metastases, preexisting active autoimmune diseases, and ongoing steroid treatment.

### Ethics statements

The trial was approved by the Ethics Committee of Sun Yat-sen University Cancer Center (B2021-207-08). All participants gave their written consent prior to being included in the study. This trial has been registered on ClinicalTrials.gov under the identifier NCT04996771.

### Treatment and assessment

The study was a dose-explored phase Ib/II trial conducted. Surufatinib monotherapy has been approved globally (300 mg qd, po) to treat NET from all sources of organs, including lungs. The initial dose of surufatinib combined with toripalimab, etoposide plus cisplatin in 3 + 3 dose escalation phase was set at 200 mg qd, po, in order to maximum the survival benefits for patients in a potential tolerable dose. In the 3 + 3 dose escalation phase, if no DLT occurred in the first 3 patients, the dose would be adjusted to 250 mg, qd, po; if DLT occurred in one of the first 3 patients, 3 more patients would be further enrolled at 200 mg; if DLT occurred in ≥ 2 patients of the first 3 patients, the dose would be adjusted to 150 mg, qd, po. DLTs were observed for the 1st cycle. The other patients received RP2D of surufatinib every 3 weeks for 4 cycles in subsequent phase II trial. All enrolled patients should also receive toripalimab (240 mg, d1, ivdrip), etoposide (100 mg/m², d1-d3, ivdrip) plus cisplatin (25 mg/m², d1-d3, ivdrip) every 3 weeks for 4 cycles and received previously same doses of surufatinib and toripalimab every 3 weeks as maintenance therapy until unacceptable toxicity or disease progression. The order of administration was as follows: toripalimab, etoposide, cisplatin, and surufatinib. The dosing regimens of toripalimab, etoposide, and cisplatin was based on the EXTENTORCH study.^13^ Surufatinib was given since the first day of therapy with no interruption based on the previously phase I study.^28^ Tumor response was assessed every 6 weeks ( ± 7 days).

### Endpoint

The primary end point was PFS. PFS was defined as the time from the first dose of either drug to radiologic PD assessed by investigator or to death from any cause. The secondary end points included ORR, DCR, DOR, and OS. ORR was defined as the proportion of complete response (CR) or partial response (PR) on at least one visit, while DCR was defined as the proportion of patients with a CR or PR or stable disease (SD). DOR was defined for patients who achieved a CR or PR as the date at which the patient’s objective status was first noted to be a CR or PR to the earliest date relapse was documented. OS was defined as the time from patient enrollment to death from any cause. AEs graded according to the Common Terminology Criteria for Adverse Events (CTCAEs) (version 5.0) were recorded throughout the study.

### Biomarker analysis

The baseline Ki-67 levels of tumor tissue and the levels of serum NSE & ProGRP were collected retrospectively to explore whether Ki-67, NSE or ProGRP would have influence on tumor shrinkage rate, PFS, and OS. The cutoff value of Ki-67 was 90%, while the cutoff of NSE or ProGRP was its median value. We also collected data from pretreatment samples of enrolled patients retrospectively to explore whether PD-L1 expression and TMB level would affect the curative effect. The PD-L1 expression was assessed using the PD-L1 immunohistochemical 22C3 pharmDx assay, then determined by the Tumor Proportion Score (TPS). TPS <1 was classified as PD-L1 negative. NGS test was performed on Gene^+^ Seq-2000 sequencing system through a panel containing 1021 cancer-associated genes or MGISEQ-2000 sequencing system through a panel containing 688 cancer-associated genes. TMB of > 10 mutations/Mb was classified as high.

### Statistical analysis

The previously reported mPFS of chemotherapy (etoposide plus cisplatin) for treatment-naïve ES-SCLC was 4.3 months.^6^ We expected the mPFS of surufatinib plus toripalimab combined with etoposide and cisplatin as first-line treatment for ES-SCLC was 7.3 months with one-sided α value (type I error) of 0.05 and statistical power (type II error) of 0.80. The sample size was calculated by PASS 15 software (version 15.0.5) in a One-Sample Logrank tests mode. Scheduled recruiting and follow-up time were both 12 months. A total of 32 patients needed to be enrolled within the scheduled accrual and follow-up to achieve 26 disease progression events in a 10% drop-out rate. Missing data were not imputed when performing statistical analyses. Efficacy analyses were done in per protocol set including all the patients who had at least one post-baseline disease assessment within 6 weeks of the first dose, while the AEs were analyzed for the safety set (SS) with all patients who received at least one cycle of the treatment. The ORR and DCR analyses were based on frequencies calculated with its two-sided 95% confidence intervals (CIs) using the Clopper-Pearson approach. The median values for PFS, OS, and DOR were analyzed with the Kaplan-Meier method, and their 95% CIs were derived by the Brookmeyer and Crowley methods respectively. Wilcoxon rank-sum test was used for the comparisons of tumor shrinkage rate stratified by different cohorts. The safety evaluation was mainly based on descriptive statistical analysis. All statistical analyses were performed using SAS software (version 9.4, SAS Institute Inc, Cary, USA).

## Supplementary information

Supplementary Materials

Dataset 1

## Data Availability

The original data supporting the results of this manuscript had been deposited in the Research Data Deposit repository (https://www.researchdata.org.cn) under the accession code (RDDA2024883468). Access to the data should be requested by the corresponding authors. Besides, processed NGS data of 8 enrolled patients was consolidated in Data S1.
