# Supplementary material for: Surufatinib plus toripalimab combined with etoposide and cisplatin as first-line treatment in advanced small-cell lung cancer patients: a phase Ib/II trial
Source: Signal Transduct Target Ther. 2024 Sep 27;9:255. doi: 10.1038/s41392-024-01974-2 (PMC11427686; doi:10.1038/s41392-024-01974-2)
Supplement: Supplementary file 1 — Supplementary Materials [file 41392_2024_1974_MOESM1_ESM.docx]

**Supplementary Materials for**

Surufatinib plus toripalimab combined with etoposide and cisplatin as first-line treatment in advanced small-cell lung cancer patients: a phase Ib/II trial

**Author(s) list**

Yaxiong Zhang^1,#^, Yan Huang^1,#^, Yunpeng Yang^1,#^, Yuanyuan Zhao^1,#^, Ting Zhou^1,#^, Gang Chen^1^, Shen Zhao^1^, Huaqiang Zhou^1^, Yuxiang Ma^2^, Shaodong Hong^1^, Hongyun Zhao^2^, Li Zhang^1,*^, Wenfeng Fang^1,*^

^#^These authors contributed equally:

Yaxiong Zhang, Yan Huang, Yunpeng Yang, Yuanyuan Zhao, Ting Zhou.

***Corresponding author:**

Wenfeng Fang, (Email: [fangwf@sysucc.org.cn](mailto:fangwf@sysucc.org.cn))

Li Zhang (Email: zhangli@sysucc.org.cn).

**This file includes:**

Figures S1 to S5

Tables S1 to S2

Caption for Data S1


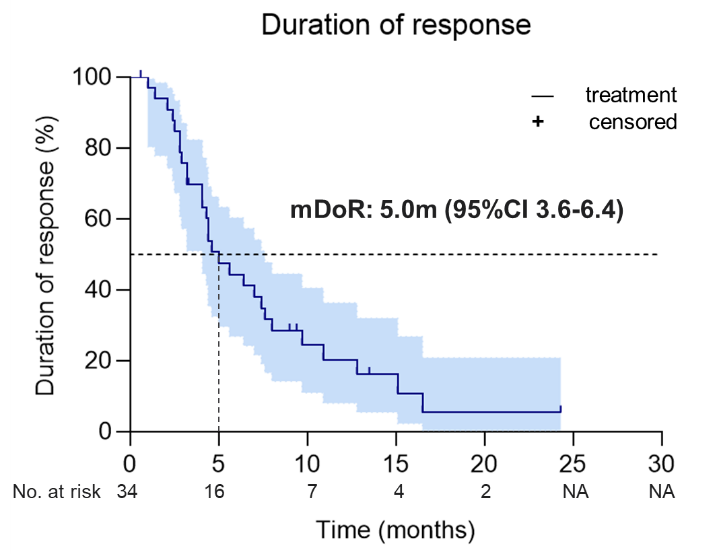

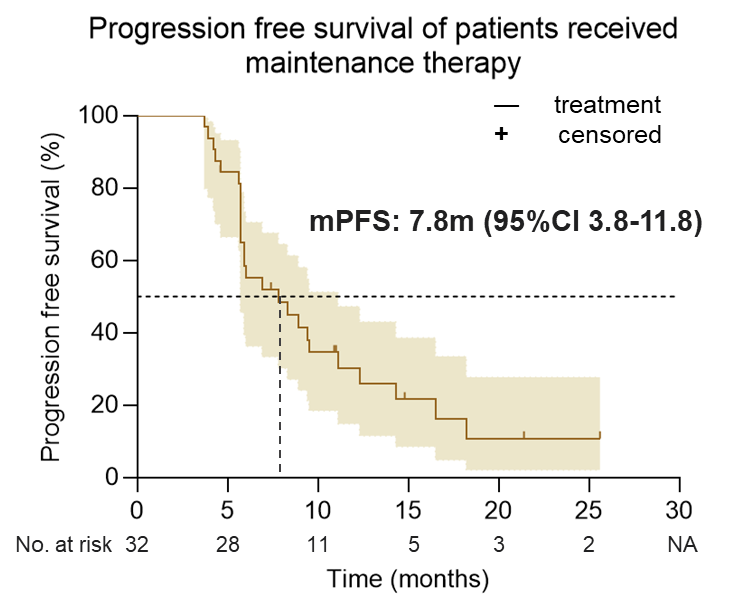


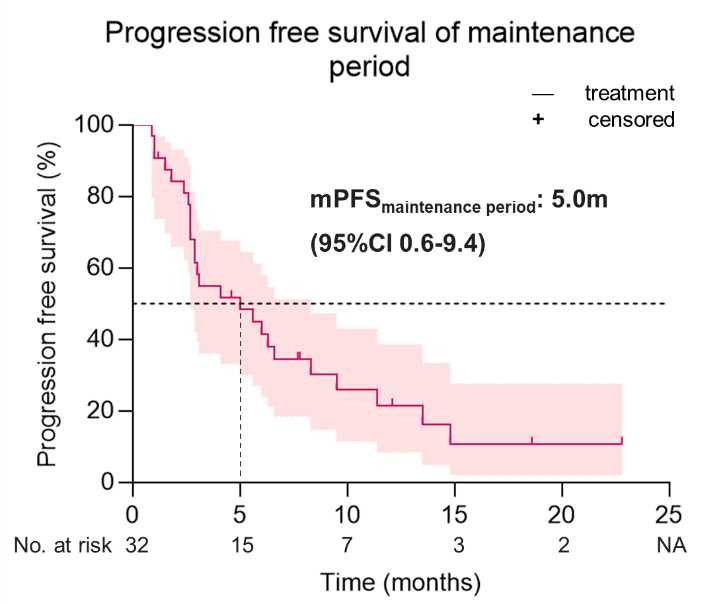


**Figures S1**. Kaplan–Meier curves of duration of response, progression-free survival of patients received maintenance therapy and progression-free survival of the maintenance period.


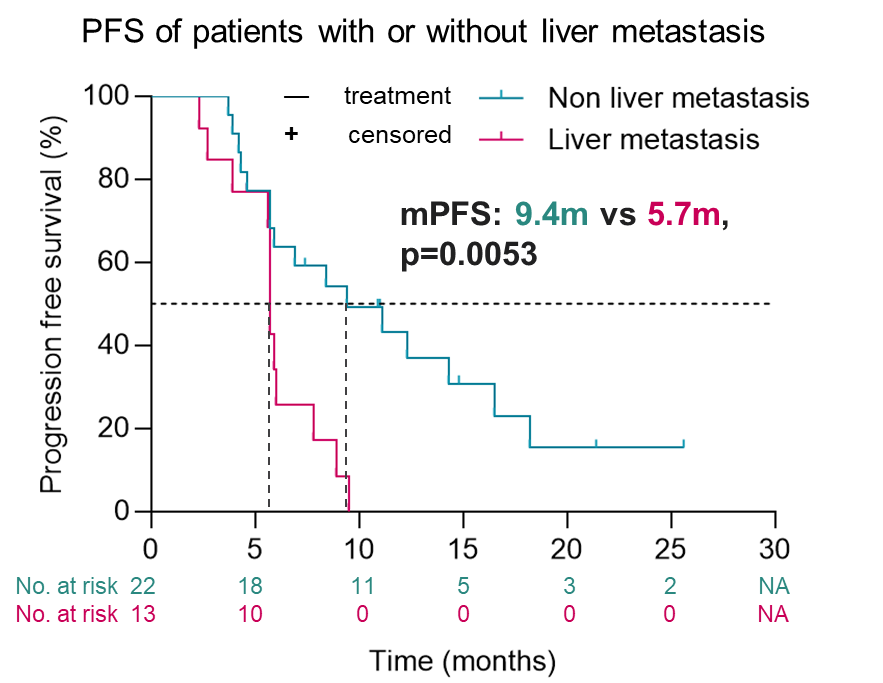


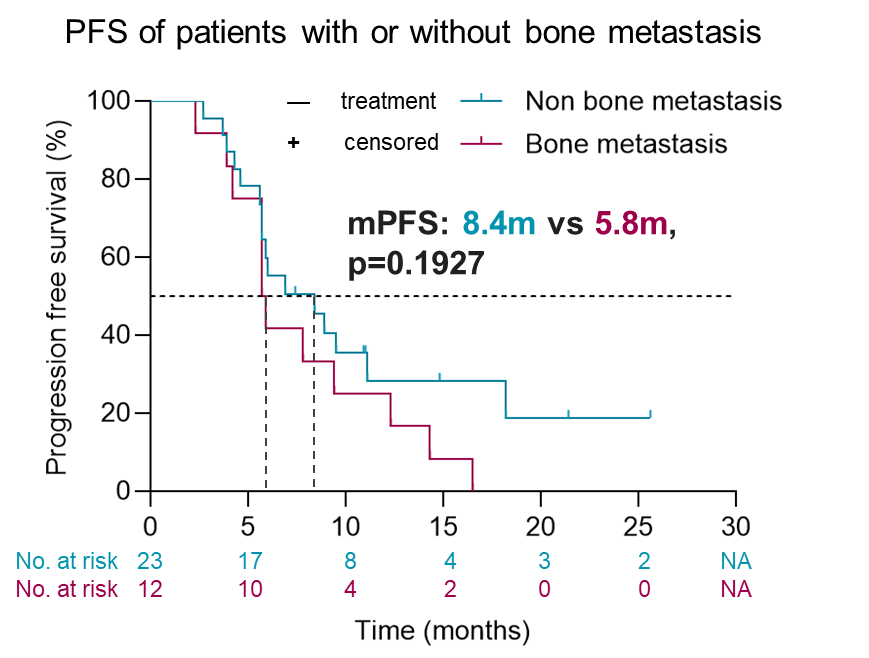


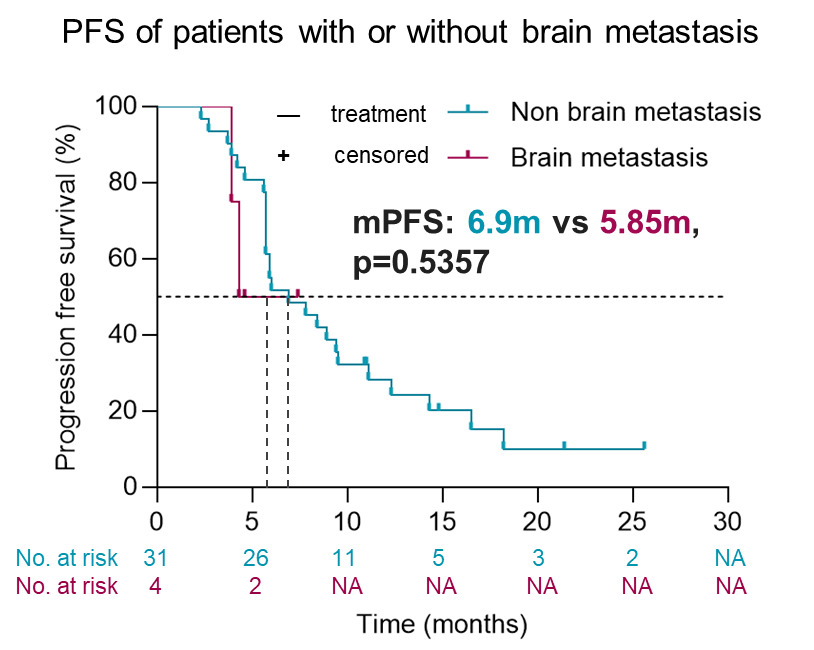


**Figure S2**. Subgroup analysis of progression-free survival stratified by different metastatic sites.


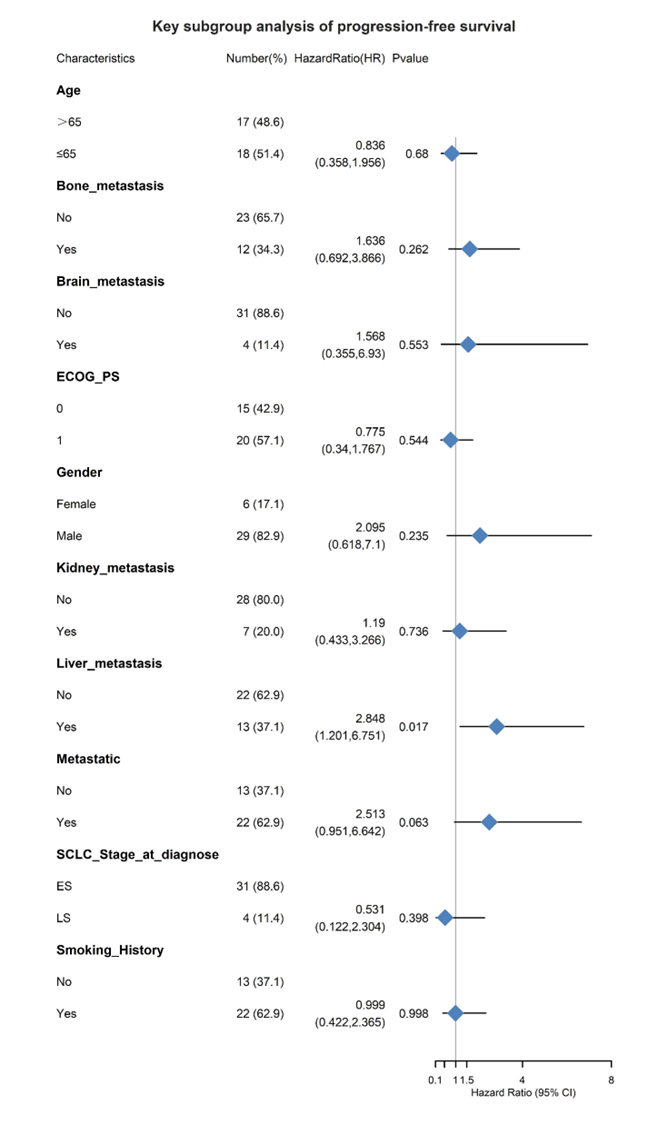


**Figure S3**. Univariate Cox regression analysis of progression-free survival.


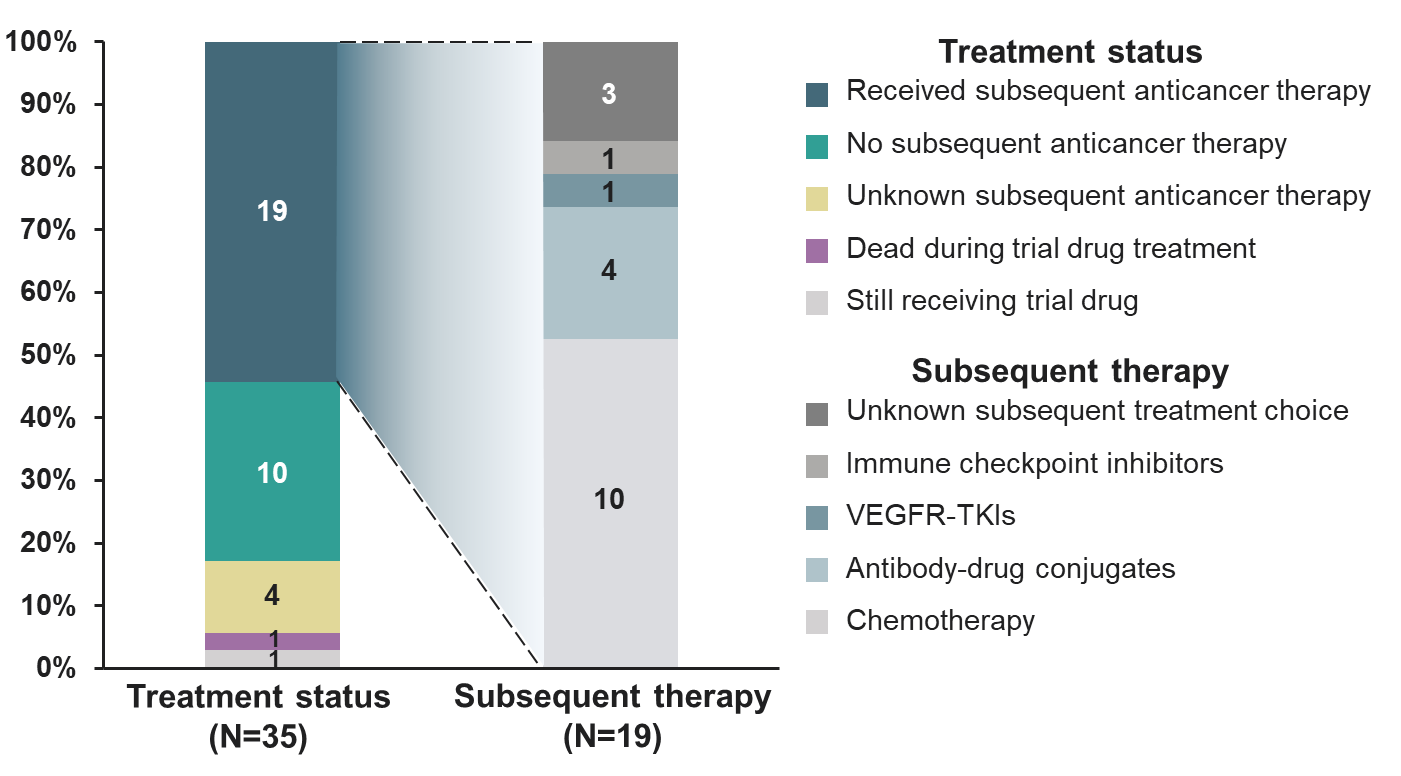


**Figure S4**. Subsequent therapies of the enrolled patients.


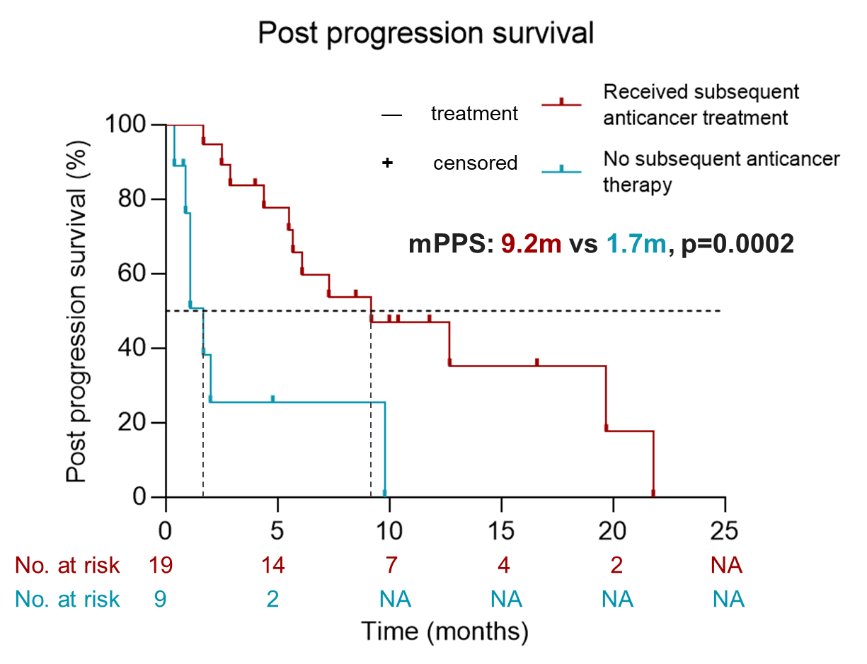


**Figure S5**. Influence of subsequent therapies on post progression survival of the enrolled patients.

**Table S1**. Tumor shrinkage rate and PFS & OS stratified by microsatellite status, TMB and PD-L1 expression at baseline.

| **Patient No.** | **MSS** | **TMB** | **PD-L1 expression** | **Tumor response (shrinkage rate, %)** | **PFS**  **(months)** | **OS**  **(months)** | **Status** |
| --- | --- | --- | --- | --- | --- | --- | --- |
| 1012 | UK | UK | TPS<1% | PR (-36.0) | 3.7 | 5.4 | Dead |
| 1028 | UK | UK | TPS<1% | PR (-63.1) | 6.0 | 6.8 | Loss of follow up |
| 1031 | UK | UK | TPS=30% | PR (-60.7) | 16.5 | 18.2 | Still alive |
| 1039 | MSS | Low (6.72Muts/Mb) | UK | PR (-30.6) | 3.9 | 4.8 | Dead |
| 1026 | MSS | Low  (7.68 Muts/Mb) | UK | PR (-37.7) | 3.9 | 5.0 | Dead |
| 1004 | MSS | Low  (7.68 Muts/Mb) | UK | PR (-59.8) | 4.3 | 10.4 | Dead |
| 1033 | MSS | Low  (7.68 Muts/Mb) | UK | PR (-59.6) | 4.6 | 4.6 | Loss of follow up |
| 1013 | MSS | Low  (6.72 Muts/Mb) | UK | PR (-39.1) | 6.9 | 14.2 | Dead |
| 1006 | MSS | Low  (4.3 Muts/Mb) | UK | PR (-60.5) | 7.4 | 27.1 | Dead |
| 1038 | MSS | High  (10.56 Muts/Mb) | UK | PR (-69.2) | 8.9 | 17.4 | Still alive |
| 1009 | MSS | High  (14.4 Muts/Mb) | UK | PR (-90.8) | 14.8 | 28.9 | Still alive |

Abbreviations:TPS tumor proportion score, MSS microsatellite stable, TMB tumor mutational burden, PR partial response, PFS progression free survival, OS overall survival, UK unknown.

**Table S2**. Serious adverse events.

| **Events, n (%)** | **All patients (n=38)** | |
| --- | --- | --- |
|  | **Any grade** | **Grade 3 or higher** |
| Incomplete intestinal obstruction | 1 (2.6) | 1 (2.6) |
| Platelet count decreased | 2 (5.2) | 2 (5.2) |
| COVID-19 infection | 1 (2.6) | 1 (2.6) |
| Transient atrial fibrillation | 1 (2.6) | 0 |
| Pleural effusion | 1 (2.6) | 0 |
| Rash | 1 (2.6) | 0 |
| Hyponatremia | 1 (2.6) | 1 (2.6) |
| Elevated serum amylase | 1 (2.6) | 1 (2.6) |
| Neutrophil count decreased | 1 (2.6) | unknown |
| Febrile neutropenia | 1 (2.6) | 1 (2.6) |
| Atrial fibrillation | 1 (2.6) | 1 (2.6) |

**Data S1**. **(separate file)** NGS data of 8 enrolled patients.
